# A Case of Granulomatosis with Polyangiitis (Wegener’s Granulomatosis) Presenting with Rapidly Progressive Glomerulonephritis

**DOI:** 10.7759/cureus.5896

**Published:** 2019-10-12

**Authors:** Md Rockyb Hasan, Md Sakibuzzaman, Tahsin Tabassum, Syed Ahmad Moosa

**Affiliations:** 1 Internal Medicine, Dhaka Medical College, Dhaka, BGD; 2 Internal Medicine, Sir Salimullah Medical College, Dhaka, BGD; 3 Virology, Bangabandhu Sheikh Mujib Medical University, Dhaka, BGD; 4 Family Medicine, Woodhaven Medical Professional Corporation, Queens Village, USA

**Keywords:** rapidly progressive glomerulonephritis, necrotizing granulomatous inflammation, lung parenchymal disease

## Abstract

Granulomatosis with polyangiitis (GPA, Wegener’s granulomatosis) presenting as rapidly progressive glomerulonephritis is not uncommon. The recognition of multisystem disease involving joints, kidney, and lung is critical for diagnosing Wegener's vasculitis. Here, we report a case study of a 52-year-old Bangladeshi man presented with a history of progressively worsening fever, recurrent cough, and hemoptysis. He developed renal failure within a month which was successfully treated with high-dose steroids, cyclophosphamide, and trimethoprim-sulfamethoxazole (TMP-SMX). Rapidly progressive glomerulonephritis can be a fulminant manifestation of GPA, in which case an immediate and aggressive treatment with pulse steroids, high-dose cyclophosphamide and TMP-SMX can be lifesaving.

## Introduction

Granulomatosis with polyangiitis (GPA, Wegener’s granulomatosis) is one of the antineutrophil cytoplasmic antibody (ANCA)-associated small vessel vasculitides involving various organs such as nasal septum, sinuses, upper respiratory tract, lungs, and kidneys. GPA is pathologically characterized by necrotizing granulomatous inflammation [1,2]. ANCA-associated small vessel vasculitides represent a major challenge in hospital admissions. Therefore, early and accurate diagnosis with aggressive treatment is essential to improve the disease outcome. In this article, we present a case of GPA with P-ANCA positive rapidly progressive glomerulonephritis. We also explore the differential diagnosis and discuss its treatment in the Department of Medicine, Dhaka Medical College Hospital, Bangladesh.

## Case presentation

**Figure 1:**
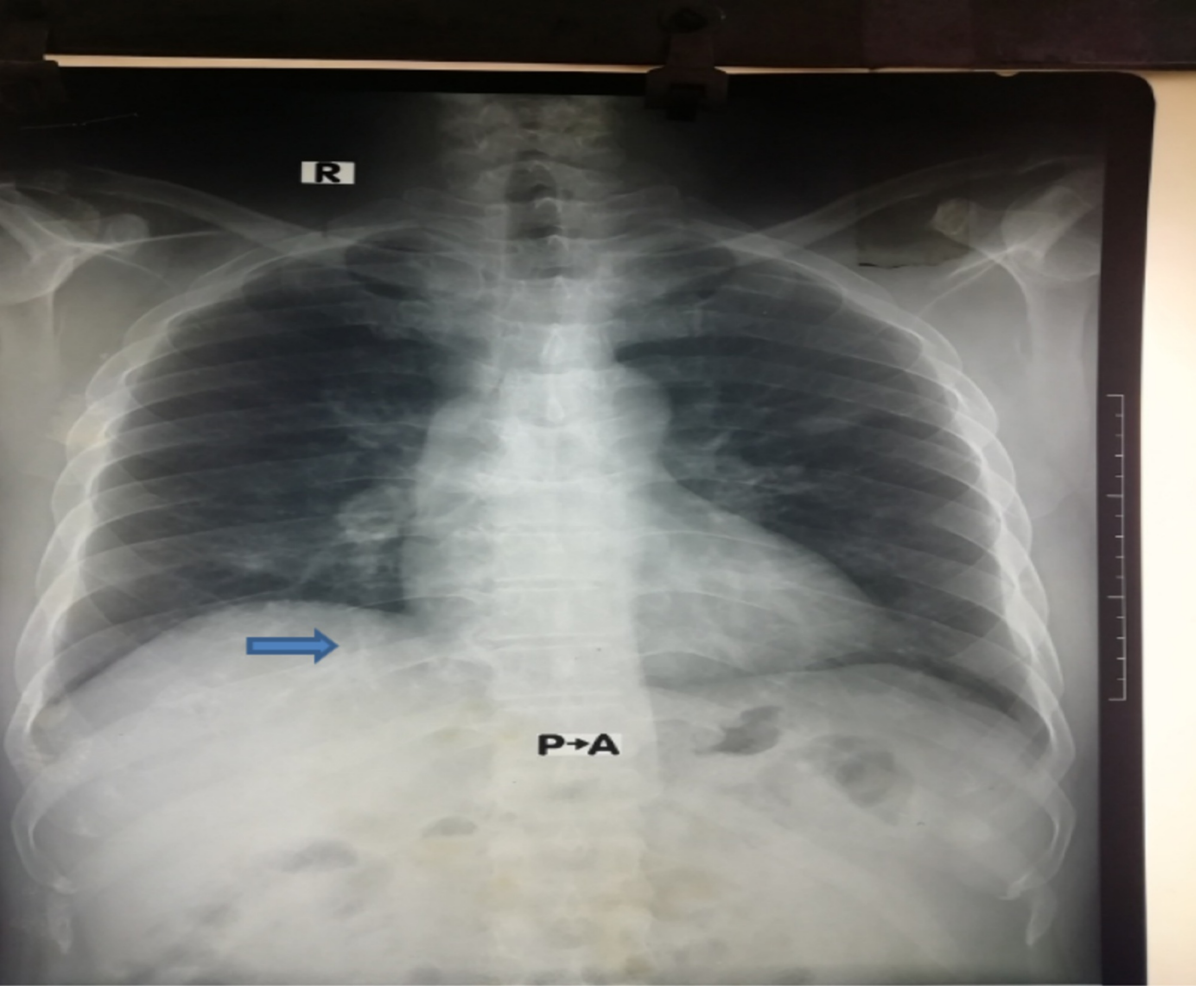
X-ray showing some reticulonodular shadows scattered all over the lung field The blue arrow is showing cavitation on right side of lung. P-A stands for posterior-anterior view of X-ray.

**Figure 2:**
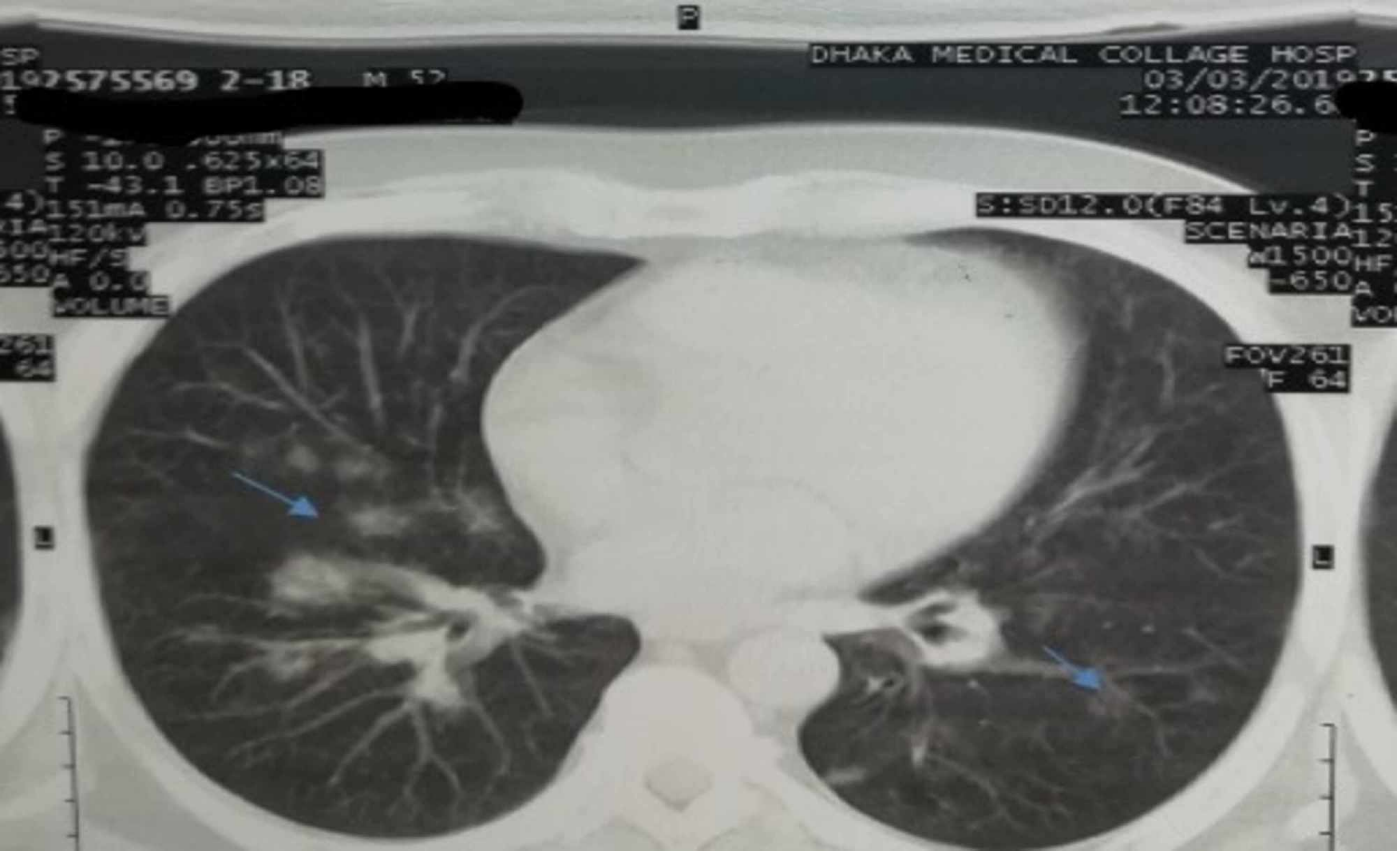
CT scan of the chest showing multiple dense nodular shadows with some cavitation involving upper and middle lobes of both lungs. The blue arrows are indicating inhomogeneous patchy opacities and cavitation throughout the lung.

A 52-year-old male resident from Dhaka, Bangladesh, admitted into Dhaka Medical College Hospital on 19th March 2019 with the complaints of fever, cough, and recurrent hemoptysis for one month. The patient was hypertensive, non-diabetic, and non-asthmatic. He also mentioned having a runny nose with sneezing which persisted for a few days for the last 10 years associated with recurrent nasal crusting. He added that he experienced multiple large and small joints pain with significant morning stiffness following an episode of chikungunya fever one year back and for that, he used to take aspirin and nonsteroidal anti-inflammatory drugs (NSAID) occasionally. On examination, his pulse was 78 beats per minute, blood pressure was 160/98 mm of Hg, the temperature was 100 degrees Fahrenheit, mild anemia and other systems examination revealed no significant abnormalities. Laboratory investigations showed hemoglobin of 8.7 gm/dl, erythrocyte sedimentation rate (ESR) of 80 mm in 1st hour, and microcytic hypochromic anemia on peripheral blood film. Routine urine examination showed plenty of red blood cells, significant proteinuria, and urinary protein creatinine ratio of 1.41. His serum creatinine was 6.2 mg/dl. His chest X-ray (Figure 1) showed some reticulonodular shadows scattered all over the lung field and CT scan of the chest (Figure 2) showed multiple dense nodular shadows with some cavitation involving upper and middle lobes of both lungs. Further investigations revealed antinuclear antibody panel (ANA) and rheumatoid factor (RF) titers to be negative, but his perinuclear (p)-ANCA autoantibody was positive at 19 U/mL. Additionally, his cytoplasmic (c)-ANCA autoantibody and anti-glomerular basement membrane (GBM) immunoglobulin titers were both negative. The result of the patient’s purified protein derivative test was negative and three acid-fast bacilli smear tests of sputum came out negative. GeneXpert was also negative for MTB. Renal biopsy showed pauci-immune deposition of antibodies, mostly IgG, in a linear pattern with crescent formation. We diagnosed this patient as ANCA-associated GPA and treated him with intravenous Methylprednisolone pulse therapy (1 gram/day) for three days followed by 60 mg oral prednisolone and 150 mg of oral azathioprine. The symptoms including respiratory and renal functions became normal within four weeks. He was advised for follow-up six weekly with complete blood count, urine routine microscopic examination and serum creatinine. Drugs were gradually lowered to 10 mg of prednisolone without repeated attack. The patient is under management of Department of Medicine, Dhaka Medical College Hospital, Bangladesh with treatment and regular follow-up in every six weeks.

## Discussion

GPA consists of necrotizing granulomatous inflammation of upper and lower respiratory tracts, rapidly progressive glomerulonephritis, and necrotizing vasculitis involving lungs and a variety of systemic organs and tissues. It is most frequently manifested as parenchymal lung disease resulting in multiple nodules and masses although they usually do not yield typical radiographic pattern like it did in our patient. The patient’s progressive multisystem complaints over a period of months of fever, cough, hemoptysis along with the elevated ESR, and anemia strongly supported the differential diagnosis of bronchogenic carcinoma, pulmonary tuberculosis or systemic vasculitis. In our differential diagnosis, after excluding bronchogenic carcinoma and pulmonary tuberculosis, we included among the systemic vasculitides, GPA, microscopic polyangiitis, Churg-Strauss syndrome, and anti-GBM or Goodpasture syndrome as well as systemic lupus erythematosus, and less likely, Behçet’s disease and rheumatoid arthritis [3]. This patient’s clinical course and his strongly positive c-ANCA with negative RF, ANA, p-ANCA and anti-GBM titers were considered diagnostic of GPA [3]. Our patient manifested many typical GPA symptoms and signs, including cough (34% on presentation), hemoptysis (18%), nasal congestion and epistaxis (11%), pulmonary infiltrate (71%), purpuric rash (13%) and renal failure (11%) [4]. Also, c-ANCA directed against PR3 is most specific for GPA. Some patients with GPA express p-ANCA specific for myeloperoxidase (MPO) as this patient was p-ANCA positive. Combining immunofluorescence and ELISA enhances the sensitivity and specificity of a diagnosis of ANCA-associated vasculitis to 96% and 98.5%, respectively. Usually, renal involvement is severe and is the leading cause of mortality [5]. Likewise, our patient’s renal function deteriorated to crescentic glomerulonephritis or rapidly progressive glomerulonephritis. However, his significant proteinuria, hematuria, and renal biopsy were also suggestive of GPA-associated glomerulonephritis. The combination of high-dose corticosteroids and cyclophosphamide is the mainstay of treatment for vasculitis and disease resistance to this combination is rare. Intravenous cyclophosphamide (0.5 g/m^2^ to 1.0 g/m^2^ body surface area) is started at the same time as pulse methylprednisolone (1 g intravenous for three days), followed by high-dose steroid treatment adjusted to response. The dose of Cyclophosphamide is given at nine repeat intervals - the first six cycles at a space of three weeks, and the last three cycles two weeks apart. Trimethoprim-sulfamethoxazole (TMP-SMX) have been found to be beneficial in patients with GPA in remission by decreasing relapse by the elimination of the microbe responsible and hence, ending the initiation of the harmful stimulus [6,7]. TMP-SMX can be given in the initial phase of GPA as well as in cases of less severe generalized GPA [8,9].

## Conclusions

GPA often presents as crescentic glomerulonephritis. GPA should be critically considered as diagnosis when a presentation involves multiple systems including joint, kidney, and lung. Lifesaving treatment of GPA is based on prompt and aggressive administration of pulse steroids, high doses of cyclophosphamide in repeat intervals, and TMP-SMX.
